# Role of Adenosine A_2A_ Receptors in Modulating Synaptic Functions and Brain Levels of BDNF: a Possible Key Mechanism in the Pathophysiology of Huntington's Disease

**DOI:** 10.1100/tsw.2010.164

**Published:** 2010-09-01

**Authors:** Maria Teresa Tebano, Alberto Martire, Valentina Chiodi, Antonella Ferrante, Patrizia Popoli

**Affiliations:** Department of Therapeutic Research and Medicine Evaluation, Istituto Superiore di Sanità, Rome, Italy

**Keywords:** adenosine A_2A_, BDNF, synaptic transmission, hippocampus, Huntington’s disease

## Abstract

In the last few years, accumulating evidence has shown the existence of an important cross-talk between adenosine A_2A_ receptors (A_2A_Rs) and brain-derived neurotrophic factor (BDNF). Not only are A_2A_Rs involved in the mechanism of transactivation of BDNF receptor TrkB, they also modulate the effect of BDNF on synaptic transmission, playing a facilitatory and permissive role. The cAMP-PKA pathway, the main transduction system operated by A_2A_Rs, is involved in such effects. Furthermore, a basal tonus of A_2A_Rs is required to allow the regulation of BDNF physiological levels in the brain, as demonstrated by the reduced protein levels measured in A_2A_Rs KO mice. The crucial role of adenosine A_2A_Rs in the maintenance of synaptic functions and BDNF levels will be reviewed here and discussed in the light of possible implications for Huntington's disease therapy, in which a joint impairment of BDNF and A_2A_Rs seems to play a pathogenetic role.

### INTRODUCTION

Neurotrophins, namely brain-derived neurotrophic factor (BDNF), nerve growth factor (NGF), neurotrophin 3 (NT-3), and neurotrophin 4/5 (NT-4/5), are small signaling molecules that play a central role in many central nervous system (CNS) functions, promoting neuronal proliferation, differentiation, and survival[1], as well as synaptic plasticity[2,3,4].

The actions of neurotrophins are mediated by two classes of cell surface receptors: tropomyosin-related kinase receptors (Trk A,B,C), members of the tyrosine kinase family, and p75 neurotrophin receptor (NTR), a member of the tumor necrosis factor receptor superfamily[5,6]. In addition to the canonical agonist-mediated receptor activation, Trk receptors can be transactivated in response to G protein-coupled receptor (GPCR) signaling[7,8,9]. This additional mechanism of Trk receptor activation is particularly relevant since, even though neuroprotective effects of neurotrophins have been described in a number of neurodegenerative diseases, neurotrophins' inability to cross the blood brain barrier makes their possible therapeutic application difficult.

Among the four neurotrophins, the actions of BDNF on central neurons have been characterized the best. BDNF has the widest distribution in the CNS, where it is mostly expressed in the cerebral cortex and the hippocampus[10,11], and it has emerged as a major regulator of synaptic plasticity, neuronal survival, and differentiation. In addition, compelling evidence suggests its possible pathogenetic role in Huntington's disease (HD), a rare and disabling genetic neurodegenerative disorder, characterized by choreic movements, psychiatric symptoms, dementia, and early death. A massive and quite selective loss of GABAergic medium-size spiny neurons (MSN) in the striatum is the distinctive feature of this pathology that, to date, remains incurable (reviewed in [12,13]).

Adenosine, a purine nucleoside present in all cells, is a fundamental neuromodulator and regulator of homeostasis in the brain. Its effects are mediated by four receptors ($A_1$, $A_{2A}$, $A_{2B}$, and $A_3$) belonging to the GPCR family[14]. Adenosine $A_{2A}$ receptors ($A_{2A}$Rs), which are highly expressed in the basal ganglia, but widespread over all the brain, play a facilitatory role on the release of different neurotransmitters and regulate excitotoxic mechanisms, exerting either neuroprotective or detrimental effects depending on the nature of the brain injury and on tight functional interactions with other receptor systems. $A_{2A}$Rs show several structural and functional characteristics that allow them to elicit different biological responses, depending on the cellular context and on the nature of the concomitant signals[15,16].

In the last few years, it has been demonstrated that adenosine $A_{2A}$R is specifically involved in the modulation of BDNF effects through different and independent mechanisms: (1) direct activation of TrkB receptors in the absence of BDNF (a process called transactivation)[17] and (2) facilitation of fast synaptic action of BDNF on hippocampal transmission[18,19].

In this paper, the crucial role of adenosine $A_{2A}$Rs in the maintenance of synaptic functions and brain levels of BDNF will be reviewed and discussed in the light of possible implications for HD therapy.

### BDNF AND SYNAPTIC TRANSMISSION

BDNF is a small dimeric protein and most of its biological effects are mediated by the receptor TrkB. The binding with BDNF results in the dimerization and autophosphorylation of the receptor that, after the activation, triggers at least three different signal transduction cascades: (1) the mitogen-activated protein kinase (MAPK) pathway, involved in differentiation and axonal growth; (2) the phosphatidylinositol 3-kinase (PI3K), a major survival pathway; and (3) the phospholipase C $\gamma$ (PLC $\gamma$) pathway, specifically involved in synaptic plasticity[20]. BDNF and TrkB receptors show widespread distribution across all the subregions of the hippocampus and an overlapping at glutamatergic synapses[21]. This finding accounts for the important role played by BDNF in synaptic transmission, since appropriate expression and level of activation of the complex BDNF-TrkB appear critical for modulating synaptic efficacy[3] and the response to excitotoxic injury[22,23].

Over the last several years, studies have suggested that BDNF, in addition to regulating neuronal survival through the traditional neurotrophic effects, also modulates synaptic transmission[24], exerting fast excitatory actions in neurons, controlling resting membrane potential and neuronal excitability, and participating in the induction of long-term changes in synaptic transmission[25,26,27,28]. In particular, in the adult hippocampus, BDNF is critically involved in the regulation of synaptic plasticity[29] and facilitates long-term potentiation (LTP), a cellular basis for information storage (for review see [4,26,30]). These effects underlie the proposed role for BDNF in learning and memory processes[31]. Furthermore, a specific BDNF-induced potentiation of excitatory synaptic transmission (termed BDNF-LTP) has been reported in the CA1[32,33], in the dentate gyrus[34,35], and in hippocampal cell cultures[36,37,38,39]. Even though the ability of BDNF to enhance synaptic transmission when directly applied to hippocampal slices appears controversial, a recent paper showed that such an effect is highly influenced by the different experimental conditions[40].

Although the mechanisms responsible for BDNF synaptic effects are not completely understood, modifications of presynaptic neurotransmitter release, rapid effects on postsynaptic ion channels, and pre- and postsynaptic N-methyl-D-aspartic acid (NMDA) receptors are known to be involved[25,41,42,43]. It is important to point out that the synaptic and neuroprotective effects of BDNF seem to be mediated by different mechanisms. In fact, ligand-induced TrkB translocation in lipid rafts is required for short-term modulation of synaptic transmission, but not for promoting neuronal survival[44,45].

### ADENOSINE

Adenosine is an important neuromodulator that is produced in the extracellular space through two different mechanisms: (1) ectonucleotidase degradation of ATP released by neurons and astrocytes, and (2) intracellular production followed by extracellular transport[46]. Adenosine plays many physiological roles, including regulation of sleep, pain, arousal, and locomotor behavior[47]. Adenosine's effects are mediated by the binding to the four GPCR subtypes: $A_1$, $A_{2A}$, $A_{2B}$, and $A_3$[14].

Adenosine receptors are ubiquitous, with almost all cell types expressing functional forms of at least one of the four known subtypes. $A_1$ and $A_{2A}$ subtypes are activated at low concentrations of extracellular adenosine (high-affinity receptors). $A_1$Rs are widely expressed in peripheral tissues[46], while in the CNS, high levels can be found in the striatum, cortex, cerebellum, and hippocampus[48]. The $A_1$Rs are responsible for the majority of the adenosine-depressant activities and their activation promotes energy sparing and protective actions within the whole body[49]. At the synaptic level, neuroprotection is directly related to the ability of $A_1$Rs to inhibit synaptic transmission during the insult, and this is most probably due to a concerted inhibitory action upon glutamate release at the presynaptic level and NMDA activation at the postsynaptic level.

The $A_{2A}$R subtype represents a key mediator of the behavioral effects of caffeine (the most widely used drug in the world), which acts as an adenosine receptor antagonist[50]. Even though the mRNA for the adenosine $A_{2A}$Rs has been found in almost all the areas of the CNS, a high density of the receptor protein occurs predominantly on neurons in the striatum (GABAergic striatopallidal projection neurons, cholinergic interneurons), in the nucleus accumbens, and olfactory tubercle[51]. To a lesser extent, $A_{2A}$Rs have been found in the hippocampus and cerebral cortex[52,53], although the populations that predominate in these areas are not identical to the "classical" striatal receptors[54].

The main second messenger pathway linked to the $A_{2A}$Rs is the activation of adenylyl cyclase, leading to intracellular cyclic adenosine monophosphate (cAMP) increase. It has been demonstrated that under physiological conditions, activation of the $A_{2A}$Rs is responsible for a tonic increase in basal cAMP levels[55,56].

$A_{2A}$R is expressed not only on neurons, but also on glial cells and seems to be critically involved in the modulation of astrocytic response to injury and inflammation[57].

$A_{2A}$Rs can be found both pre- and postsynaptically. At a presynaptic level, activation of $A_{2A}$Rs facilitates glutamate release so that $A_{2A}$R antagonists are regarded as promising neuroprotective drugs, in conditions in which excitoxicity plays a pathogenic role[58]. In contrast, it has been found that the blockade of $A_{2A}$R does not reduce or may even potentiate the effects elicited by direct NMDA receptor activation both in the hippocampus[59] and in the striatum[60,61,62]. Thus, it would seem that $A_{2A}$R activation differentially influences excitotoxic mechanisms, exerting harmful effects by its ability to increase extracellular glutamate levels at the presynaptic site, but also potentially beneficial effects by modulating NMDA receptor activity at the postsynaptic site[62].

However, besides their direct pre- and postsynaptic actions on neuron receptors, $A_{2A}$Rs are primarily involved in triggering or modulating the activation/inactivation of other neurotransmitters or neuromodulators throughout a sophisticated cross-talk either at the transducing system level[63] or following-up receptor oligomerization[64,65].

In brief, $A_{2A}$R sites bind with distinct neurotransmitter receptors to form divergent receptor entities at different synaptic levels. In fact, at the presynaptic site, the $A_{2A}$R forms dimers with $A_1$R[66,67] and type 1 cannabinoid receptor (CB1R)[68], while at the postsynaptic site it associates with the dopamine D2 receptor ($D_2$R)[69,70,71] and/or type 5 metabotropic glutamate receptor (mGlu5R)[72,73] and possibly CB1R[74]. However, at the striatal level, multiple interactions among $A_{2A}$, $D_2$, mGlu5, and CB1 receptors have been described at the biochemical and behavioral level[64,65,68], suggesting the possible existence of higher-order oligomers (for reviews [75,76,77,78]).

### $A_{2A}$RS AND BDNF CROSS-TALK

#### Transactivation and Neuroprotection

The first link between BDNF and $A_{2A}$Rs was provided in 2001 by Lee and Chao[17] with the demonstration that activation of the tyrosine kinase Trk receptor can also occur via a GPCR mechanism, without involvement of neurotrophins (a mechanism called transactivation). Specifically, it has been shown that activation of TrkA in PC12 cells and TrkB in hippocampal neurons could be obtained in the absence of neurotrophins by treatment with adenosine. These effects were reproduced by using the agonist CGS 21680 and were counteracted by the antagonist ZM 241385, indicating the involvement of the $A_{2A}$R subtype. The transactivation, recently also reported *in vivo*[79], requires long-term incubation with GPCR agonists and receptor internalization[80]. Apparently, $A_{2A}$Rs are able to modulate TrkB neuroprotective function within lipid rafts and nonlipid raft membranes, possibly through the cAMP-independent pathway involving the Src family kinase[81], thus improving neuronal survival directly transactivating the protective TrkB-Akt pathway.

Since in conditions in which there is an increase of adenosine release and thus of $A_{2A}$R activation, an increase of neurotrophin release also occurs, the interaction between these two modulators may become particularly relevant. An enhancement of extracellular adenosine levels can be reached during depolarization or high neuronal activity that causes, together with an increase of neurotransmitter release, an increase of ATP release. Both conditions favor the activation of $A_{2A}$Rs. Likewise, on the other hand, it is widely accepted that depolarization triggers a facilitatory action of BDNF on synaptic potentiation[82]. Moreover, neuronal activity regulates the transcription of the BDNF gene, the transport of BDNF mRNA and protein into dendrites, and the secretion of the BDNF protein[29].

#### Synaptic Transmission: Facilitatory and Permissive role of $A_{2A}$Rs

As previously reported (see above), BDNF is involved in synaptic transmission. The hypothesis that adenosine $A_{2A}$R activation could represent a crucial requisite for the functioning of neurotrophic receptors at synapses was previously explored by Diogenes and coworkers in 2004 by electrophysiological studies in the CA1 area of rat hippocampal slices[18]. These authors observed that, while in hippocampal slices from infant rats, BDNF alone was devoid of effect. It becomes able to enhance hippocampal transmission when the $A_{2A}$R was activated by CGS 21680, or when adenosine extracellular levels were increased by 5-iodotubercidin or by inducing a presynaptic depolarization by a pulse of high $K^+$[83]. The excitatory action of BDNF was blocked by the TrkB receptor inhibitor K252A, by the adenosine $A_{2A}$R antagonist ZM 241385, and by the protein kinase A inhibitor H-89. Therefore, they concluded that presynaptic activity-dependent release of adenosine, through activation of $A_{2A}$Rs, facilitates BDNF modulation of synaptic transmission at hippocampal synapses. A similar positive interaction has been confirmed at the neuromuscular junction[84]. Furthermore, when hippocampal slices from adult rats were used, BDNF was able to increase the excitatory postsynaptic field potential (fEPSP) by itself, an effect that was abolished by ZM 241385[85].

The hypothesis that $A_{2A}$Rs play a major role in regulating BDNF functions has been strengthened by further observations from our group[62] showing that the tonic activation of $A_{2A}$Rs is required for BDNF-mediated synaptic effects. Specifically, we demonstrated that in hippocampal slices from WT mice, application of BDNF by itself increased the slope of fEPSPs, an index of synaptic facilitation. This effect was abolished by the pharmacological blockade of $A_{2A}$Rs (by two different $A_{2A}$R antagonists, ZM 241385 and MSX3) as well as by the genetic deletion of the receptor. However, the inability of BDNF to facilitate synaptic transmission in $A_{2A}$R KO mice did not depend on a reduced density of TrkB receptors since the expression of the receptor was not altered in these mice. Thus, while the earlier results of Diogenes et al.[18] indicate a facilitatory action of hippocampal $A_{2A}$Rs towards BDNF synaptic effects, our study provided evidence of a permissive role played by $A_{2A}$Rs.

It is worth mentioning that in our experimental conditions, the coapplication of BDNF and CGS 21680 did not facilitate BDNF effects. The lack of potentiating effects of CGS 21680 suggests that the state of activation of $A_{2A}$Rs ensured by endogenous adenosine could be already maximal and sufficient to manifest BDNF effects. In agreement with this finding, it has been reported[86] that the activation of $A_{2A}$R failed to increase the BDNF-induced facilitation of LTP in the CA1 region of the hippocampus. When the endogenous adenosine was depleted by adenosine deaminase (ADA), and thereby $A_{2A}$Rs were not tonically activated, CGS 21680 was able to facilitate the action of BDNF on LTP[86], thus demonstrating that the selective activation of adenosine $A_{2A}$Rs is critically involved in BDNF modulation of CA1 LTP.

As with stimulation of $A_{2A}$Rs by CGS 21680, the genetic overexpression of these receptors also does not result in the facilitation of BDNF-induced effects. In fact, in hippocampal slices originating from $A_{2A}$R-overexpressing rats[87], BDNF failed to increase the synaptic transmission (Martire and Chiodi, unpublished data).

### Mechanisms of $A_{2A}$ and BDNF Cross-Talk

The main second messenger pathway linked to the $A_{2A}$Rs involves adenylyl cyclase/cAMP-PKA, and the influence of $A_{2A}$R on BDNF-induced synaptic effects seems to be mediated by the activation of this pathway. On the other hand, even though the cAMP-PKA pathway is not a downstream effector in the BDNF-TrkB signaling cascade[88], cAMP plays a crucial role in controlling BDNF effects in the brain. In fact, at neuromuscular junctions, it has been demonstrated that while the activation of cAMP signaling was ineffective in modifying synaptic efficacy, it enhanced the potentiating effect of BDNF[89]. Furthermore, the blockade of cAMP signaling abolished the facilitation of BDNF-induced potentiation of synaptic transmission, thus suggesting that cAMP could act as a “gate” for BDNF signaling and synaptic actions. Selective inhibitors of PKA prevented both the synaptic effects of BDNF in hippocampal slices of WT mice[62] and the enhancement of LTP caused by BDNF when $A_{2A}$Rs were activated by CGS 21680 in an adenosine-depleted background[86]. To further support these findings, the cAMP-PKA pathway has also been implicated in modulating BDNF gene transcription[90] and release[91,92] (Fig. 1).

### Influence of Age

After the paper by Kang and Schuman in 1995[32] showing that BDNF enhances synaptic transmission in the rat hippocampus, in several following studies, BDNF failed to influence basal synaptic transmission in the hippocampus[18,93,94]. Other than the difference in experimental condition and animal species, it is the different age of the animals that most probably accounts for this discrepancy. Interestingly, the same group who previously reported that BDNF was devoid of action on synaptic transmission by itself[18], reported in a subsequent study[85] that BDNF increased the fEPSP slope recorded in slices from the hippocampus of young adult and aged rats, but not in infant animals. The selective $A_{2A}$R antagonist ZM 241385 prevented the excitatory effect of BDNF. In order to trigger a facilitatory action of

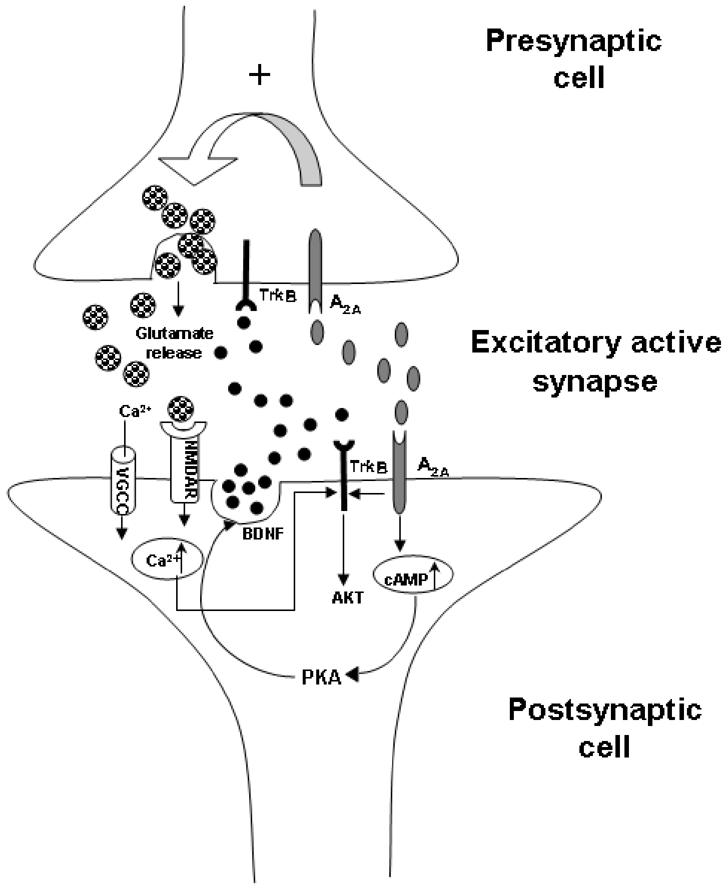

**FIGURE 1.** Scheme of the signaling involved in BDNF synaptic effects: role of $A_{2A}$Rs. High neuronal activity triggers secretion of BDNF, as well as enhancement of extracellular adenosine levels and activation of $A_{2A}$Rs that, in turn, stimulate glutamate release. At the postsynaptic level, $Ca^{2+}$ influx through voltage-gated channels (VGCC) or NMDA receptors (NMDAR) triggers BDNF secretion. $A_{2A}$Rs activate adenylyl cyclase, which leads to production of cAMP and activation of PKA that "gates " BDNF secretion. TrkB activation by BDNF stimulates different pathways involved in neurotrophic and survival effects. Activation of $A_{2A}$Rs can modulate TrkB neuroprotective functions by directly transactivating the protective TrkB-Akt pathway. BDNF facilitates synaptic potentiation either through activation of the PLC $\gamma$ pathway, specifically involved in synaptic plasticity, and/or by modulating the expression and trafficking of NMDARs.

BDNF in these animals, it was necessary to increase the extracellular levels of adenosine by inhibiting adenosine kinase[18,84] or by using a high-frequency stimulation protocol[86]. The authors concluded that age-related changes in the density of TrkB and $A_{2A}$Rs, and their degree of activation, may account for the age-related synaptic effects of BDNF. Thus, in aged animals, the decrease in BDNF-LTP is due to an impaired TrkB density and signaling[35,95] that could be partially compensated by a higher density of $A_{2A}$Rs[85]. Therefore, it is possible that the facilitatory effect of BDNF on synaptic transmission depends on the presence of balanced levels of $A_{2A}$ and TrkB receptors. Even though controversial findings regarding modifications of BDNF and its receptors in aging have been reported[96], it has been recently found that a long-lasting treatment (from 6 to 18 months of age) with caffeine (a preferential $A_{2A}$R antagonist) prevented the age-related change in BDNF and TrkB hippocampal immunocontent and cognition decline[97].

A lot of evidence indicates that BDNF and its receptor have an important role in aging (reviewed in [96]) and in age-related alterations, such as learning and memory processes[98]. However, the idea that reduced hippocampal levels of BDNF have to be necessarily associated with memory deficits should be reconsidered, since intact spatial learning and memory have been reported in transgenic mice with reduced BDNF[99]. In addition, as both BDNF effects and levels significantly reduced in the hippocampus of $A_{2A}$R KO mice, one might expect a certain degree of memory impairment as a result. Instead, an improvement in spatial memory was reported in $A_{2A}$R KO mice[100]. Since adenosine $A_{2A}$Rs negatively influence learning and memory processes (see also the recent finding of an impairment in working memory in rats overexpressing $A_{2A}$Rs[87]), it is conceivable that the "beneficial" influence of $A_{2A}$R deletion overcomes the "negative" influence of reduced BDNF levels.

### Regulation of BDNF Protein Levels

The mechanisms involved in BDNF secretion and release have been extensively studied in the past years (for complete reviews, see [101,102,103,104]).

Briefly, BDNF (both the precursor and the mature form) is contained in secretory vesicles present in both axon terminals (presynaptic site) and dendrites (postsynaptic site), mainly of glutamatergic neurons[29,101,105,106]. BDNF can be secreted from either postsynaptic spines or presynaptic terminals. BDNF levels are regulated in postnatal development, in part by activity-dependent mechanisms[107]. Whatever the method used to increase synaptic activity (depolarization, high-frequency stimulation, etc.), different studies (in neurons and cell lines) demonstrated that BDNF secretion is dependent on $Ca^{2+}$ influx through NMDA receptors or voltage-gated $Ca^{2+}$ channels[108], and on mobilization of $Ca^{2+}$ from intracellular stores[109] (Fig. 1). As previously mentioned, the cAMP-PKA pathway also participates in BDNF release. In hippocampal neurons, basal levels of PKA activity seem to be sufficient to allow BDNF secretion[92]. Indeed, while the cAMP-PKA signaling inhibitor Rp-cAMP-S significantly inhibited and delayed BDNF secretion, elevation of intracellular cAMP levels by the PKA activator 8-Br-cAMP neither induced nor facilitated BDNF secretion. Since the cAMP-PKA pathway is the main transduction system operated by $A_{2A}$Rs, this finding is in line with the observation that in $A_{2A}$R KO mice, the reduced functional ability of BDNF in facilitating synaptic transmission correlated with the reduction of the BDNF levels compared with the WT littermates[19]. Even though changes in release cannot entirely account for the significant reduction in BDNF levels we found in $A_{2A}$R KO mice, we can speculate that a normal state of activation of $A_{2A}$Rs exerts a kind of "permissive" role on the maintenance of normal BDNF levels. The $A_{2A}$R permissive role was confirmed by the reduction of BDNF levels in naïve mice treated *in vivo* with the selective $A_{2A}$ antagonist ZM 241385. Furthermore, similar to the synaptic effect, no increase in the hippocampal levels of BDNF were observed in WT mice treated i.p. with the $A_{2A}$R agonist CGS 21680[110], thus suggesting that the endogenous state of activation of $A_{2A}$Rs and PKA are adequate to sustain a normal BDNF secretion.

## ROLE OF $A_{2A}$R-BDNF INTERACTION IN NEURODEGENERATIVE DISEASES

Changes in neurotrophin levels or in their effects have been implicated in different neurodegenerative diseases, such as Alzheimer's disease, Parkinson's disease, Huntington's disease, amyotrophic lateral sclerosis (ALS), and in mood disorders such as depression and schizophrenia[111,112,113]. The inability of these molecules to cross the blood brain barrier hampers their therapeutic use, prompting the design of efficient delivery strategies and/or alternative approaches, even invasive. Thus, the evidence that the stimulation of $A_{2A}$Rs triggers or facilitates BDNF effects could open new possibilities for the exploitation of this neurotrophin for therapeutic uses. However, as discussed in detail in a recent review[114], the pharmacological intricacy of $A_{2A}$R has to be taken into account, since its activation or blockade can be neuroprotective depending on time windows of neurodegenerative diseases and the nature of neuronal damage.

Although only limited evidence is available so far, some findings suggest that the $A_{2A}$R-BDNF cross-talk may play a role in ALS. Interestingly, in contrast with the reports that demonstrate a beneficial effect of neurotrophins in neurodegeneration, limited evidence is available showing that BDNF signal activation could also play a detrimental role. Indeed, it has been demonstrated that the susceptibility of motor neurons to excitotoxic insults is promoted by BDNF[115] and that preventing TrkB activation protected motor neurons from excitotoxic insult[116]. More recently, in spinal cord culture (grown in the presence of a cocktail of trophic factors including BDNF), chronic application (for 2–4 days) of $A_{2A}$R antagonist protected motor neurons from excitotoxic insult with kainic acid, inducing an inhibition of the Trk activation as shown by reduction in Trk phosphorylation[81]. Coimmunoprecipitation analysis showed that $A_{2A}$ and TrkB receptors are colocalized on motor neurons, and Src family tyrosin kinases (SFKs) are also involved in $A_{2A}$R-BDNF cross-talk. In fact, TrkB, adenosine $A_{2A}$R, and SFKs associate into complexes in lipid raft, and disruption of lipid rafts by cholesterol depletion blocks the ability of BDNF to render motor neurons vulnerable to insult. These results further emphasize that changes in TrkB activation can be a function of adenosinergic neurotransmission and that $A_{2A}$Rs may be targeted to “drive” BDNF effects.

### Role of $A_{2A}$-BDNF Interaction in Huntington’s Disease

A specific link between an impairment in BDNF function and the pathogenesis of Huntington’s disease (HD) has been demonstrated by Cattaneo’s group (for review, see [13,117]). HD is an inherited neurodegenerative disease caused by a mutation in the protein huntingtin, and characterized by marked cortical and striatal degeneration. BDNF is colocalized with huntingtin in cortical neurons that project to the striatum, and most striatal BDNF is produced in the cerebral cortex and anterogradely transported into vesicles along the corticostriatal afferents[118]. Cortical production and striatal delivery of BDNF thus depends on the presence of normal huntingtin (for review, [12]). In both animal models of HD and in patients, a decreased huntingtin-mediated BDNF gene transcription has been reported, since the normal protein regulates the activity of the BDNF promoter[119,120,121] by inhibiting the repressor element 1/neuron-restrictive silencer element (RE1-NRSE) that is located in BDNF promoter exon II. Inactivation of the RE1-NRSE in BDNF leads to increased mRNA transcription and protein production in the cortex, which is then made available to the striatal targets via the corticostriatal afferents. Wild-type huntingtin could also facilitate vesicular BDNF transport from the cortex to the striatum[12,122]. Thus, it would seem that a reduced striatal BDNF availability makes neurons more susceptible to degeneration and, in fact, its exogenous administration allows striatal neurons to survive from excitotoxin-induced neurodegeneration[123]. BDNF is reduced in the HD human brain and also in some models of the disease[117]. However, in R6/2 mice, a most widely used transgenic model of HD[124], even though a reduction in BDNF mRNA has been reported[125], basal protein levels were not significantly altered with respect to WT[110,126]. Mutated huntingtin altering the BDNF trophic support towards the striatum may preferentially affect the function of the subpopulation of MSNs expressing $A_{2A}$Rs and a lot of evidence (reviewed in [127,128]) indicates a possible pathogenetic involvement of striatal $A_{2A}$Rs in HD. For instance, $A_{2A}$Rs are localized on GABAergic enkephalin neurons that degenerate early in HD, so that their expression is reduced in the basal ganglia of HD patients at a very early stage[129]; $A_{2A}$Rs are able to stimulate glutamate outflow and excitotoxic mechanisms seem to be involved in HD[130,131]; $A_{2A}$R expression and underlying signaling systems undergo profound changes in cellular and animal models of HD[128,132].

On the basis of the above observations, a possible neuroprotective role of adenosine $A_{2A}$R antagonist has been envisaged. On the other hand, however, the inhibitory effects exerted by $A_{2A}$R blockade on BDNF levels and functions may limit the therapeutic potential of $A_{2A}$R antagonists.

Indeed, in quinolinic acid (QA)–lesioned rats, a pathogenetic model of HD-like striatal degeneration[133], and in a transgenic model of HD (R6/2 mice) during the early symptomatic phase of the disease (5–8 weeks), the systemic administration of $A_{2A}$R antagonist SCH 58261 reduced striatal

BDNF levels[110]. Furthermore, in electrophysiological experiments in corticostriatal slices from R6/2 mice, the blockade of $A_{2A}$Rs prevented BDNF-induced attenuation of NMDA toxicity (Martire et al., manuscript in preparation). Worthy of note, however, when the treatment with the $A_{2A}$R antagonist was performed in a late phase of the disease progression (8–11 weeks), SCH 58261 did not modify BDNF protein levels in the striatum[134]. Since expression changes and functional alterations of $A_{2A}$Rs occur as a consequence of the disease[129,135,136,137,138], it could be that the different effect observed when performing the treatment in different periods is due to the fact that the receptor is present in a different functional state according to the stage of the disease. Indeed it has been demonstrated that in symptomatic R6/2 mice, the treatment with the selective $A_{2A}$R agonist CGS 21680 reduced the NMDA-induced toxicity in corticostriatal slices[139] and, *in vivo*, delayed progressive deterioration of motor coordination, reduced the size of intranuclear inclusions[138], and modulated the subunit composition of NMDA receptors[140]. Even though the levels of BDNF protein were unchanged, the reduced expression of the receptor TrkB was increased in the cortex of R6/2 mice at the end of treatment with CGS 21680 (Ferrante et al., unpublished results). These findings suggest that in a frankly symptomatic phase of the disease, $A_{2A}$R agonists may become neuroprotective and that $A_{2A}$R-BDNF cross-talk might play a role in such an effect. This is in line with the view that the complex profile of $A_{2A}$R influences its relevance as a therapeutic target[141].

### CONCLUSIONS

Adenosine $A_{2A}$R plays a major role in regulating BDNF functions. Its activity favors, at least in part, the prosurvival function of BDNF, its synaptic activity, and its tissue availability, thus confirming it to be an important tuner of brain activity. Although this could open up new strategies in neuronal dysfunctions in which a pathogenetic role of BDNF has been shown, any "therapeutic" approach based on $A_{2A}$Rs will have to take into account the very complex pharmacological effects of such receptors.
